# Sydenham's chorea in a family with Huntington's disease: case report and review of the literature

**DOI:** 10.1590/S1516-31802011000400011

**Published:** 2011-05-05

**Authors:** Rita Santos-Silva, Susana Corujeira, Ana Filipe Almeida, Sofia Granja, Cludia Moura, Ins Azevedo, Miguel Leo, Ana Maia

**Affiliations:** I MD. Resident in Pediatrics, Department of Pediatrics, Hospital So Joo, Porto, Portugal.; II MD. Resident in Pediatric Cardiology, Department of Pediatric Cardiology, Hospital So Joo, Porto, Portugal.; III MD. Consultant Pediatric Cardiologist, Department of Pediatric Cardiology, Hospital So Joo, Porto, Portugal.; IV MD, PhD. Associate Professor of Pediatrics of the School of Medicine of Porto University; Pediatric Pulmonology Unit, Department of Pediatrics, Hospital So Joo, Porto, Portugal.; V MD. Consultant Neurologist and Geneticist, Neuropediatric Unit, Department of Pediatrics, Hospital So Joo, Porto, Portugal.; VI MD. Consultant Pediatrician, General Pediatrics Unit, Department of Pediatrics, Hospital So Joo, Porto, Portugal.

**Keywords:** Chorea, Huntington disease, Rheumatic fever, Antistreptolysin, Genetic counseling, Coria, Doena de Huntington, Febre reumtica, Antiestreptolisina, Aconselhamento gentico

## Abstract

**CONTEXT:**

Sydenham's chorea affects almost 30% of patients with acute rheumatic fever. It is more frequent in females and is rare in the first decade of life, and genetic vulnerability underlies it. Because of easy access to antibiotics, it is now rare in so-called developed countries.

**CASE REPORT:**

A 6-year-old boy with a family history of Huntington's disease, who was the only child of an unscreened and asymptomatic mother, was brought for a consultation because of migratory arthralgia, depressed mood, and rapid, abrupt and unintentional movements of his right arm and leg, that had evolved over a three-week period. On physical examination, he presented a grade III/VI systolic heart murmur and right-side choreic movements, giving rise to a deficit of active mobilization. Laboratory tests revealed elevated erythrocyte sedimentation rate (63 mm/h), C-reactive protein (25 mg/l) and antistreptolysin O titer (1,824 U/ml). Cardiovascular evaluation showed mild aortic insufficiency, moderate mitral insufficiency and a prolonged PR interval. A clinical diagnosis of Sydenham's chorea/acute rheumatic fever was made, and therapy consisting of penicillin, haloperidol, captopril and furosemide was instituted, with excellent results.

**CONCLUSION:**

In developed countries, Sydenham's chorea seems forgotten and, because of this, little is known about its clinical course and controversy surrounds the therapeutic options available. This occurrence of rheumatic chorea in a family with Huntington's disease highlights the importance of the differential diagnosis for the different forms of chorea.

## RESUMO

**CONTEXTO:**

A coreia de Sydenham surge em cerca de 30% dos casos de febre reumtica aguda. mais frequente no sexo feminino, rara na primeira dcada de vida e tem por base uma vulnerabilidade gentica. Devido ao fcil acesso aos antibiticos, uma doena rara atualmente nos pases ditos desenvolvidos.

**RELATO DO CASO:**

Criana de seis anos, sexo masculino, com histria familiar de coreia de Huntington, nico filho de me assintomtica e no rastreada, foi trazido consulta por artralgias migratrias, humor deprimido e movimentos rpidos, abruptos e no intencionais dos membros superior e inferior direitos, com trs semanas de evoluo. Ao exame fsico, apresentava um sopro cardaco sistlico grau III/VI, e foram presenciados movimentos coreicos direita, condicionando um dfice de mobilizao activa. Os exames laboratoriais mostraram aumento da velocidade de sedimentao (63 mm/h), protena C-reativa (25 mg/L) e ttulo de antiestreptolisina O (1.824 U/mL). O exame cardiovascular revelou insuficincia artica ligeira e insuficincia mitral moderada e aumento do intervalo PR. Foi feito o diagnstico de coreia de Sydenham/febre reumtica aguda, tendo sido instituda teraputica com penicilina, haloperidol, captopril e furosemida, com excelente resultado.

**CONCLUSO:**

Nos pases desenvolvidos, a coreia de Sydenham parece esquecida e, por isso, pouco se sabe quanto ao seu curso clnico e as opes teraputicas disponveis so controversas. A ocorrncia de um caso de coreia reumtica numa famlia com doena de Huntington reala a importncia do diagnstico diferencial das diferentes formas de coreia.

## INTRODUCTION

Chorea is a manifestation in a large number of disorders, both acquired and inherited, including metabolic, infectious, inflammatory and neurodegenerative diseases. Sydenham's chorea (SC) is the most common form of childhood chorea, while Huntington's disease (HD) accounts for the majority of adult-onset cases.^1^

## CASE REPORT

The case of a six-year-old boy who was previously healthy, but had a family history of Huntington's disease (five relatives affected, of whom two had the juvenile form of the disease) is presented here. His mother had not been screened but was asymptomatic.

He was brought to the outpatient clinic of a tertiary-care hospital in Portugal because of migratory arthralgia (without arthritis), asthenia, depressed mood and rapid, abrupt and unintentional movements of his right arm and leg (leading to severe incapacity to eat and write). The symptoms had begun three weeks earlier and he had a history of febrile syndrome, which had occurred approximately one month before the onset of his symptoms. He had not received any specific treatment and there was no history of drug use or medication allergies.

On physical examination, he presented a grade III/VI systolic murmur at the left sternal border and choreic movements in both right limbs.

Laboratory tests revealed that his complete blood cell count and blood chemistry panels were normal, but he presented elevated erythrocyte sedimentation rate (63 mm/h) and C-reactive protein (25 mg/l). The antistreptolysin O titer was 1824 U/ml and the antineuronal antibody test was negative. The cardiac evaluation showed mild aortic insufficiency and moderate mitral insufficiency, and a prolonged PR interval.

Other tests performed presented normal results, including brain magnetic resonance imaging (MRI), autoimmunity evaluation (anticardiolipin antibodies, antinuclear antibodies and complement levels), viral serological tests (human immunodeficiency virus, parvovirus B19, cytomegalovirus, Epstein-Barr virus and hepatitis C and B viruses), venereal disease research laboratory test (VDRL), blood culture and thyroid function.

The clinical picture of migratory arthralgia, rheumatic carditis and hemichorea made a diagnosis of Sydenham's chorea/acute rheumatic fever very likely. The patient was treated with benzathine penicillin (1,200,000 U), haloperidol (0.5 mg per day on the first three days and then 1 mg twice a day), captopril (12.5 mg three times a day) and furosemide (10 mg three times a day). He was discharged five days after the beginning of the treatment, with improved mood and better control over the choreic movements. One month later, he was back to his baseline behavior and the choreic movements and hypotonia had practically disappeared.

## DISCUSSION

Rheumatic fever (RF) is a nonsuppurative complication of group A beta-hemolytic streptococcal sore throat. Although rare in developed countries, RF is still present and should not be neglected. The incidence of this disease in the United States is approximately 0.5-2 cases per 100,000 population per year.^2^ Statistics concerning the incidence of this disorder in Portugal are not available. Sydenham's chorea (SC) may be present in almost 30% of patients with RF. The female-to-male ratio is 2:1, with familial predisposition, and the age of presentation is usually between 5 and 15 years.^3^

The main features of SC are behavioral changes (depression, anxiety, personality changes and emotional lability), hypotonia and purposeless movements that are aggravated by stress and absent during sleep. In 20% of these patients, only one side of the body is affected (so-called hemichorea), as in our case.^4-6^

RF diagnosed before the age of 10 years may be very different, because younger children are more likely to present arthritis and carditis but less likely to present chorea.^3,6^ However, in our case, all three of these occurred simultaneously, and the neurological manifestations were the most disabling of them.

The diagnosis of SC continues to be determined on clinical grounds, since no laboratory or imaging test can be considered to be confirmatory. Absence of a history or serological markers of prior streptococcal infection does not rule out the diagnosis of SC. Antineuronal antibodies have been found in the cerebrospinal fluid of 90% of patients with SC, and some studies have shown abnormal findings on MRI.^4^ In our case, antineuronal antibodies tested negative and MRI was normal, but there was a very high antistreptolysin O titer and there was a history of a prolonged febrile syndrome prior to the onset of symptoms that might correspond to the streptococcal infection.

SC resolves spontaneously in three to six months and, because of this, treatment should be limited to patients with severe chorea. Chorea may be controlled using either dopaminergic blockers (haloperidol or pimozide) or anticonvulsants (valproic acid and carbamazepine). Valproic acid has been recommended as the first-line treatment for SC.^7-9^ Penicillin should be used for all patients, including those with chorea alone.^10^ In the presence of antineuronal antibodies, intravenous immunoglobulin and plasma exchange are thought to be effective.^8,9^ Steroid therapy has been used in cases of severe carditis or whenever the neurological symptoms do not respond to dopaminergic blockers.^8,9^ In this particular case, we used haloperidol, with excellent results and no adverse effects, and thus without the need of any additional therapy.

Secondary prophylaxis with intramuscular benzathine penicillin every four weeks is indicated after RF to prevent further damage to cardiac valves. According to the American Heart Association, patients with RF without carditis should receive prophylactic antibiotics for five years or until aged 21; patients with RF with carditis but no valve disease should receive prophylactic antibiotics for 10 years or until 21 years of age; and patients with RF with persistent valve disease, as was the case of our patient, should receive prophylactic antibiotics *ad infinitum* or at least until 40 years of age.^10^

The prognosis for SC cases is usually good, but about 20% of patients experience recurrences within two years after the initial attack. The risk of developing psychiatric illness in adulthood, namely obsessive-compulsive disorder, is still a matter of controversy.^4^ In our case, there were no significant doubts concerning the diagnosis of SC because of the presence of carditis, migratory arthralgia, chorea and increased antistreptolysin O titer. However, other causes of chorea were considered in the differential diagnosis. First of all, other immune-mediated types of chorea, such as lupus chorea (LC), were taken into consideration. Although central nervous system involvement in cases of lupus erythematosus is common (70%), LC has been reported in less than 2% of the patients and it usually appears at a later stage of the disease.^11^ In our case, the autoimmunity evaluation was normal and there were no other manifestations of lupus, including no abnormal findings on MRI.

Another differential diagnosis to be considered is benign hereditary chorea (BHC), an autosomal dominant disorder characterized by generalized choreic movements, which usually begins in early childhood. Its severity is variable, but this condition is non-progressive and tends to disappear during adolescence, and intellectual function is typically normal.^11^ In our case, there was no family history of BHC; the patient had hemichorea (as opposed to the generalized choreic movements typical of BHC); there was an obvious cardiac involvement; and the erythrocyte sedimentation rate and antistreptolysin levels were elevated.

Curiously, we were dealing with a family with a history of Huntington's disease (HD). As far as we know, this is the first case of rheumatic chorea in a family with Huntington's disease. We carried out a systematic analysis of the indexed articles published and we only found a few reviews concerning the two subjects and some case series of "chorea". There were no cases like ours published in the main databases (Table 1).

**Table 1.** Results from reviewing the medical databases regarding the association between Sydenham's chorea and Huntington's disease

Data	Search Strategy	Results
PubMed	"Sydenham's chorea" AND "Huntington's disease", or "Rheumatic chorea" AND "Huntington's disease"	9 reviews 6 case series 2 case reports 1 clinical trial 1 experimental study 1 case-control study
	"Sydenham's chorea" AND "Family with Huntington's disease"	2 case series 2 reviews
Lilacs	"Sydenham" AND "Huntington"	1 review 1 case series
Embase	"Sydenham chorea" AND "Huntington disease"	1 review

HD is an autosomal dominant disorder with complete penetrance but age dependence, and it usually begins in the fourth decade of life. Some cases have been reported in young children, but the juvenile-onset form (Westphal variant) of chorea is usually rare and the typical presentation is a "Parkinson-like" syndrome, with rigidity, ataxia, seizures, cognitive decline and a tendency towards insanity and suicide.^11-13^ This diagnosis was ruled out in the light of the neurological manifestations and the presence of carditis. Even though a predictive genetic test is available, there is an international ethical consensus that considers that presymptomatic diagnosing of adult-onset disorders that do not have any preventive or curative treatment should only be requested or performed after obtaining fully informed consent and expression of will from capable individuals who are no longer minors.^14^ In our case, taking into account the family history (maternal grandfather affected by HD and the mother's refusal to undergo the presymptomatic diagnosis), genetic counseling would rely on a probabilistic approach, since the theoretical risk of developing HD in our patient was 25%.
